# A Crop Water Stress Index for Hazelnuts Using Low-Cost Infrared Thermometers

**DOI:** 10.3390/s24237764

**Published:** 2024-12-04

**Authors:** Dalyn McCauley, Sadie Keller, Kody Transue, Nik Wiman, Lloyd Nackley

**Affiliations:** 1 North Willamette Research and Extension Center, Oregon State University, Aurora, OR 97002, USA; dalyn.mccauley@oregonstate.edu (D.M.); nik.wiman@oregonstate.edu (N.W.); 2 Department of Horticulture, College of Agricultural Sciences, Oregon State University, Corvallis, OR 97333, USA

**Keywords:** climate change, drought, filbert, orchard, IRT, remote sensing

## Abstract

Incorporating data-driven technologies into agriculture presents a promising approach to optimizing crop production, especially in regions dependent on irrigation, where escalating heat waves and droughts driven by climate change pose increasing challenges. Recent advancements in sensor technology have introduced diverse methods for assessing irrigation needs, including meteorological sensors for calculating reference evapotranspiration, belowground sensors for measuring plant available water, and plant sensors for direct water status measurements. Among these, infrared thermometry stands out as a non-destructive remote sensing method for monitoring transpiration, with significant potential for integration into drone- or satellite-based models. This study applies infrared thermometry to develop a crop water stress index (CWSI) model for European hazelnuts (*Corylus avellana*), a key crop in Oregon, the leading hazelnut-producing state in the United States. Utilizing low-cost, open-source infrared thermometers and data loggers, we aim to provide hazelnut farmers with a practical tool for improving irrigation efficiency and enhancing yields. The CWSI model was validated against plant water status metrics such as stem water potential and gas exchange measurements. Our results show that when stem water potential is below −6 bar, the CWSI remains under 0.2, indicating low plant stress, with corresponding leaf conductance rates ranging between 0.1 and 0.4 mol m^2^ s^−1^. Additionally, un-irrigated hazelnuts were stressed (CWSI > 0.2) from mid-July through the end of the season, while irrigated plants remained unstressed. The findings suggest that farmers can adopt a leaf conductance threshold of 0.2 mol m^2^ s^−1^ or a water potential threshold of −6 bar for irrigation management. This research introduces a new CWSI model for hazelnuts and highlights the potential of low-cost technology to improve agricultural monitoring and decision-making.

## 1. Introduction

In the era of Agriculture 4.0, farmland is fertile ground for the integration of advanced digital technologies to innovate and improve crop production [1]. Sensors play a central role in this evolution, because sensing devices hold the potential for increasing the understanding of the biophysical environment that is driving plant and pest life-cycles [2]. In agricultural settings, sensing devices can gather data on various environmental parameters such as temperature, humidity, soil composition, and plant health, providing farmers with invaluable insights into their fields’ conditions [3,4]. The term “sensors” encompasses a diverse suite of technologies that can be stand-alone devices, like soil moisture probes, or multi-spectral platforms integrated onto terrestrial-based weather stations, low-altitude UASs, and global orbiting satellites. Central to the sensor-driven revolution in agriculture is sensing to enhance irrigation management, where sensors are leveraged to optimize water use efficiency [5]. Through the application of artificial intelligence and the Internet of Things (IoT), farmers can make data-driven decisions to precisely deliver water resources where and when they are needed most, mitigating water wastage and maximizing agricultural productivity [6]. Sensor-controlled irrigation represents a pivotal advancement in sustainable agriculture [7] and a critical aspect of Agriculture 4.0.

Advances in sensor technologies have led to a variety of methods for determining irrigation requirements. For instance, meteorological sensor-suites can calculate reference evapotranspiration (ET_r_), which provides a measure of weather-related demand for crop water use [8,9]. Belowground sensors can measure water volume or tension to calculate plant available water [10,11,12]. Plant sensors can provide direct measurements of water status through metrics such as stem water potential, leaf gas exchange, and sap flow measurements [13]. Infrared thermometry can be used to indirectly monitor plant water use because of the evaporative cooling effect of transpiration, which cools leaves to below the ambient air temperature [14,15]. Infrared thermometry is of particular interest because it is a non-destructive remote sensing method of monitoring transpiration that has great potential to be incorporated into drone- or satellite-based remote sensing models. For example, canopy temperature is used to model a crop water stress index (CWSI), which can be used as a tool for assessing plant water status and for scheduling crop irrigation [6,16,17]. Sensor-based technologies, like the CWSI, when combined with knowledge of crop physiology, can provide farmers with the information needed to trigger irrigation at the optimal time and avoid conditions that may lead to crop stress.

The basic principle behind the CWSI is that the transpiration rate of a plant is directly related to the temperature of the canopy. As the plant experiences water stress, its stomata close to conserve water, which reduces transpiration and leads to an increase in leaf temperature. The CWSI is calculated by comparing the actual canopy temperature to the theoretical temperature of a well-watered plant under the same atmospheric conditions. The CWSI is normalized by the upper and lower temperature threshold values for non-transpiring and fully transpiring canopies [18,19]. The resulting index ranges from 0 to 1, with higher values indicating greater water stress. The development of CWSI models has been validated in a variety of perennial crops, including apples [17], *prunus* [20,21,22], wine grapes [23], olives [24,25], and citrus [26]. While high-quality infrared thermometers and meteorological sensors are necessary to develop accurate models in a research setting, deploying and maintaining high-quality infrared thermometers and meteorological sensors at scale is economically infeasible for most farmers. The cost and maintenance associated with commercial sensing systems make publicly managed agricultural weather networks an attractive alternative option, with a low-cost and open-source weather station (LOCOS) being another attractive alternative option [27].

In contemporary agriculture, regional weather data across the United States are abundantly available, yet their integration into agricultural decision-making processes remains limited [28,29]. Furthermore, the efficacy of utilizing regional weather networks to accurately reflect site-specific conditions remains uncertain because of the variability between different on-farm microsites, especially in high-value horticultural systems, like vineyards and orchards, where slopes and aspects can be greater than in field crop systems. Consequently, farmers of high-value horticultural crops are driven to customize weather monitoring designs to suit their specific farm needs and practices, which is crucial for the success of on-farm decision-support tools [30]. The deployment of multiple on-farm weather stations holds promise, as it allows for a finer-grained understanding of weather conditions within and between fields. The benefits of managing agricultural operations at sub-field spatial scales have been underscored in the recent literature [31,32,33,34], necessitating the development and integration of distributed sensor systems and data integration platforms [35,36,37,38]. Emerging low-cost alternatives hold promise in mitigating cost barriers while maintaining performance levels comparable to commercial instruments [39]. Similarly, low-cost weather stations built on open-source platforms and embedded computer systems have demonstrated viability [25,27,39,40]. Yet, there are few evaluations of low-cost and open-source weather stations. To address this gap, we present the development and evaluation of a LOCOS, tailored to meet the needs of hazelnut (*Corylus avellana* L.) orchards.

Irrigation decision-support for hazelnuts is critically important because hazelnuts are a rapidly expanding, high-value, understudied crop with global economic significance that are most commonly grown in perennially droughty areas of the world like Turkey, Italy and Spain, Georgia and Azerbaijan, the USA, recently expanded Chile, South Africa, and Australia [41]. Oregon is the largest hazelnut-producing state in the United States, with an annual production value exceeding USD 132 million in 2020. Most new hazelnut orchards in Oregon are now using drip irrigation, and relatively little is known about hazelnut water requirements [42,43] compared to those of other tree nuts such as almonds [44,45,46]. Therefore, developing a CWSI model for hazelnuts using low-cost open-source infrared thermometers and data loggers can provide farmers with a tool to monitor crop water status, improve irrigation efficiency, and ultimately increase hazelnut yields. The goal of this study is to develop and validate a CWSI model for hazelnuts using LOCOS infrared thermometers and data loggers, and validate it against measured plant water status metrics, including stem water potential and leaf gas exchange measurements.

## 2. Materials and Methods

### 2.1. Theory

The CWSI is defined in Equation (1), where $T_{c}$ is the measured canopy temperature, and $T_{a}$ is the measured ambient air temperature. The lower ($T_{LL}$) and upper ($T_{UL}$) canopy temperature thresholds represent the temperature of a well-watered canopy transpiring at potential and the temperature of the canopy of a non-transpiring tree.

Equation (1): Crop Water Stress Index

(1) $$CWSI=\frac{{(T}_{c}-T_{a})-T_{LL}}{T_{UL}-T_{LL}}$$

In this study, $T_{C}$, $T_{LL}$, and $T_{a}$ are measured directly. Estimates for $T_{UL}$ were obtained using energy balance principles and an empirical model. Idso et al. [7] introduced the non-water-stressed baseline (NWSB), which shows that in well-watered crops, the difference between canopy temperature and air temperature, also known as leaf temperature depression (LTD), is linearly related to the vapor pressure deficit (VPD) for a variety of crops (Equation (2)).

Equation (2): The non-water-stressed baseline

(2) $$T_{c}-T_{a}=a+b\left(VPD\right)$$

Jackson et al. [8] provided a theoretical basis for the CWSI by using an energy balance equation to solve for the canopy–air temperature difference at the plant–atmosphere interface (Equation (3)), where $R_{n}$ is radiation (long- and shortwave), ρ is the density of air, $C_{p}$ is the specific heat capacity of air, γ is the psychrometric constant, ∆ is the slope of the saturation vapor pressure vs. the temperature curve, $r_{a}$ is the aerodynamic resistance, and $r_{c}$ is the canopy resistance. Aerodynamic resistance relates to the role of canopy aerodynamic friction in mediating evaporation and heat dissipation. Canopy resistance relates to the role of bulk stomatal conductance in transpiration from leaves.

Equation (3): The non-water-stressed baseline

(3) $$T_{c}-T_{a}=\frac{r_{a}R_{n}}{{\rho C}_{p}}*\frac{\gamma \left(1+\frac{r_{c}}{r_{a}}\right)}{∆+\gamma (1+\frac{r_{c}}{r_{a}})}-\frac{e_{s}-e_{a}}{∆+\gamma \left(1+\frac{r_{c}}{r_{a}}\right)}$$

For a non-transpiring canopy, canopy resistance $r_{c}$ increases without bounds ($r_{c}$→ ∞) (Equation (4)), providing an estimate of $T_{UL}$.

Equation (4): Temperature of a non-transpiring canopy

(4) $$T_{UL}-T_{a}=\frac{r_{a}*R_{n}}{\bar{\rho }c_{p}}$$

To solve for $T_{UL}$, the aerodynamic and canopy resistance terms must be known. A methodology that combines the empirical NWSB and the energy balance equation to derive the aerodynamic and canopy resistance terms can be achieved by equating Equation (2) and Equation (3), where $VPD=e_{s}-e_{a}$, and substituting the regression coefficients from the NWSB and the weather values averaged over the duration of canopy temperature measurement, $\bar{\rho }$, $\bar{R_{n}}$, $\bar{∆}$, and $\bar{r_{ap}}$, for the average aerodynamic resistance ($\bar{r_{ap}}$) (Equation (5)) and canopy resistance ($\bar{r_{cp}}$) (Equation (6)) of a canopy transpiring at potential. Substituting $\bar{r_{ap}}$ and $\bar{R_{n}}$ into Equation (4) provides an estimate of $T_{UL}$. This method has been used by several authors and with good results [47,48,49].

Equation (5): Aerodynamic resistance

(5) $$\bar{r_{ap}}=\frac{\bar{\rho }c_{p}a}{\bar{R_{n}}b\begin{array}{c} \bar{\left(\bar{∆}+\frac{1}{b}\right)} \end{array}}$$

Equation (6): Canopy resistance

(6) $$\bar{r_{cp}}=-\bar{r_{ap}}\left[\frac{∆+\frac{1}{b}}{\gamma }+1\right]$$

### 2.2. Experiment

The experiment was conducted from 25 June 2021 to 14 September 2021 on a 1-hectare hazelnut orchard at Oregon State University (North Willamette Research and Extension Center, Aurora, OR. 45.278581, −122.755672). The orchard contained cv. ‘Jefferson’ hazelnuts [50] planted using 3 × 9 m spacing. The hazelnut trees were irrigated using pressure-compensated drip emitters (1.89 LPH flow rate; Netafim USA, Fresno, CA, USA) spaced 0.5 m away from either side of each trunk and attached to 12.7 mm diameter polyethylene tubing. Each row contained two separate polyethylene tubes, allowing irrigation to be independently controlled for individual trees within the row based on their assigned irrigation treatment group. Throughout the experiment, leaks in the irrigation system were checked daily by research assistants. Visual inspections were performed to identify any punctures, typically resulting from animal chewing. Fortunately, there were no leaks detected in the irrigation system during the experiment.

The weather station located at the center of the orchard monitored various meteorological parameters essential for irrigation management. It measured incoming solar radiation using a pyranometer (LI-200SZ, Pessl Instruments, Weiz, Austria), wind speed using a cup and vane anemometer (IM512CD, Pessl Instruments, Weiz, Austria), and air temperature and humidity using a sensor (HYT221, Pessl Instruments, Weiz, Austria). These measurements provided valuable data for calculating reference evapotranspiration (ET0) and informing irrigation scheduling decisions. ET0, reference evapotranspiration, was computed daily from the weather station located in the center of the experimental orchard using Equation (3) from the FAO-56 Penman–Monteith reference evapotranspiration method [1]. To date, there are no crop coefficients (Kc) available for this hazelnut variety, and in general, there is a lack of studies on crop coefficients for any hazelnut variety. Therefore, we used ET0 as the baseline reference for irrigation scheduling, with 150% of ET0 being considered as ET0+ for the high irrigation treatment compared with dryland irrigation. Irrigation was controlled at three levels: well-watered trees received excess water by weekly irrigation at 150% of local potential evapotranspiration (ET0), moderate treatment trees were irrigated weekly at 100% of ET0, and dryland treatment trees were not irrigated. Irrigation rates were calculated accordingly from the previous week’s cumulative ET0 and applied by manually opening irrigation valves and allowing them to run for the appropriate time.

**Figure 1:**
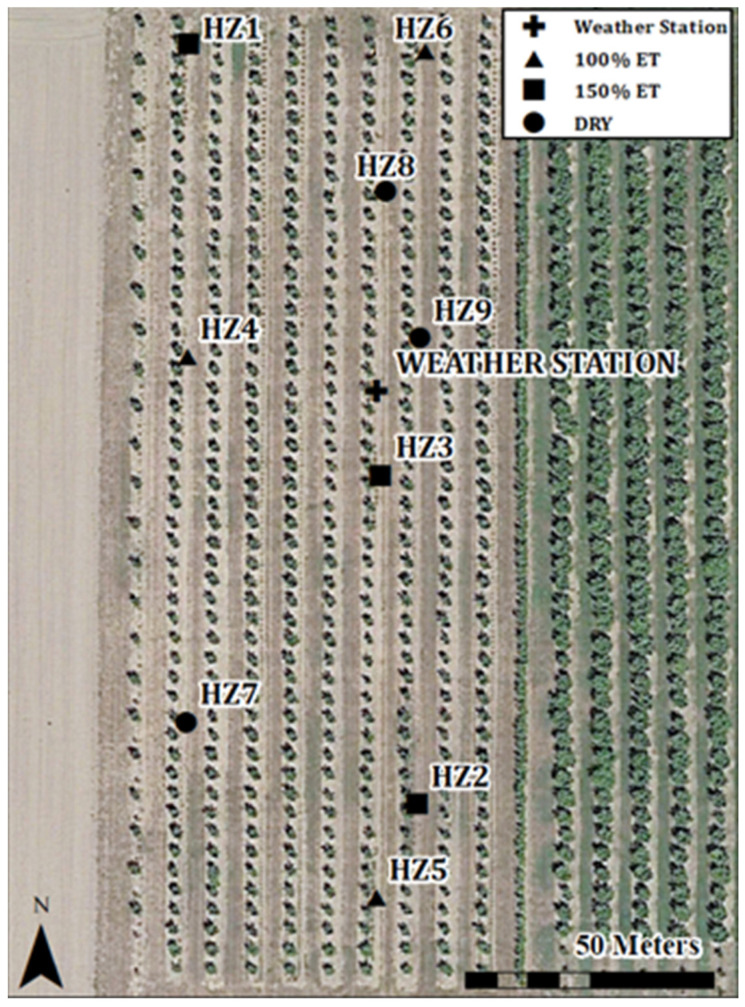
Placement of low-cost open-source (LOCO) infrared thermometers in a 1-hectare ‘Jefferson’ hazelnut orchard at Oregon State University’s North Willamette Research and Extension Center (Aurora, OR; 45.278581, −122.755672). Trees were spaced 3 × 9 m apart, and nine LOCO sensors (labeled HZ) were deployed, with symbols indicating the irrigation treatment assigned to each monitoring location.

Near-canopy weather and temperature data were recorded by a LOCOS built by our research team. The LOCOS incorporates an Arduino MEGA 2560 microcontroller (Arduino MEGA 2560; Arduino Inc., Ivrea, Italy) coupled with an Adafruit data logging shield (PN 1141, Adafruit Industries, New York City, NY, USA) to establish an embedded computer system for recording sensor data. The programming of the Arduino MEGA 2560 board was facilitated using the open-source Arduino IDE software (Arduino IDE 1.8.7, Arduino Inc., Ivrea, Italy). The data logging shield includes an SD card reader and a real-time clock (RTC) to synchronize data reading and writing processes. The total estimated cost of the electrical components required for constructing and powering the data logger system is approximately USD 150. Additionally, LOCOS were equipped with a temperature and relative humidity sensor (SHT-31 Sensirion, Basel, Switzerland) housed within a radiation shield, along with an infrared thermometer (IRT) (MLX90614, Melexis, Gent, Belgium) to measure apparent canopy temperature. Three LOCOSs were placed in each treatment, for a total of three sample trees per treatment (Figure 1). The positioning of the LOCOS was deliberate, with the infrared thermometer (IRT) placed on the north side of the tree at a height of 1.3 m from the ground, approximately in the middle of the tree canopy. This placement strategy was chosen for several reasons. Firstly, the north side of the tree provided a more stable and holistic measure of environmental conditions. In the northern hemisphere, where the experiment was conducted, the sun predominantly shines from the southern aspect. Consequently, the south side of the tree experienced greater variability in response to sun flecks and changing weather conditions, leading to potentially noisier data. Conversely, the north side presented a more conservative and representative measure of overall plant responses, as it was shielded from direct sunlight and experienced less fluctuation due to sun exposure. By positioning the LOCOS on the north side, we aimed to capture more reliable and consistent environmental data conducive to our research objectives. An essential step in thermography temperature measurement is excluding regions outside the crop canopy, such as soil or branches, as including them could yield misleading results when calculating the CWSI. The IRTs were positioned 12–17 cm from the nearest mature leaf, which resulted in a spot diameter between 24 and 34 cm with a 90° field of view. The LOCOS logged environmental data at five-minute intervals.

### 2.3. IRT Calibration

The nine IRTs were calibrated prior to deployment with a blackbody infrared calibrator (EN-37, HGH Infrared Systems, Igny, France). The blackbody temperature was manually stepped up from 25 °C to 50 °C by 5-degree increments every hour. The IRT data were logged in five-minute intervals, providing 12 data points per set-point target temperature. The root mean square error (RMSE) and the coefficient of determination (R^2^) were used to assess the accuracy of the IRT. IRT temperature-reading instability caused by changes in ambient air temperature was assessed by comparing the IRT reading of the target temperature (T_IRT_) and the set-point temperature of the blackbody (T_BB_) over a range of ambient air temperatures. The target temperature was set to 30 °C, and the IRT recorded temperature in five min intervals for 18 h (16:00–10:00) while the ambient temperature changed diurnally, ranging from 15 to 30 °C.

Emissivity correction was applied to the canopy temperature data collected in the field to account for the downwelling thermal infrared radiation from atmospheric radiance [15]. The correction was applied following the framework presented in [51]. The IRTs were positioned no more than 17 cm from the canopy. At this distance, the attenuation of thermal infrared radiation by the air column between the IRT and the canopy was assumed to be negligible, allowing the atmospheric transmission term to be set to 1. The resulting emissivity correction equation is presented in Equation (7).

Equation (7): Emissivity Correction

(7) $$T_{canopy}=\sqrt[{4}]{\frac{{T_{IRT}}^{4}-\left(1-\varepsilon_{canopy}\right)*\varepsilon_{sky}*{T_{sky}}^{4}}{\varepsilon_{canopy}}}$$

Here, $T_{canopy}$(K) is the actual kinetic temperature of the canopy, $T_{IRT}$ (K) is the apparent temperature of the canopy measured by the IRT, $T_{sky}$ (K) is the brightness temperature of the sky, $\varepsilon_{canopy}$ is the emissivity of the canopy, assumed to be 0.98 [52], and $\varepsilon_{sky}$ is the estimated emissivity of the sky. $T_{sky}$ can be approximated from the air temperature at the surface if the sky emissivity is known [53] using Equation (8). The emissivity of the sky varies between 0 and 1 depending on cloud cover. The emissivity of the sky can be approximated by modeling the clear sky emissivity ($\varepsilon_{clear}$) and applying a cloud correction factor based on the cloud conditions of the day [54].

Several methods for estimating $\varepsilon_{clear}$ have been evaluated in the literature [53,55]. In this study, the framework presented in [56] was used for its simplicity; it models the clear sky emissivity based on the vapor pressure of water ($P_{w},hPa$) at the surface (see Equation (11)). The cloud correction factor is calculated as the ratio of the solar radiation at the surface (R, MJ m^−2^ day^−1^) measured by the pyranometer in the orchard to the clear sky solar radiation, $(R_{SO}$, MJ m^−2^ day^−1^), which is calculated using the FAO-56 Irrigation and Drainage Paper method [57]. The actual sky emissivity ($\varepsilon_{sky}$) under current sky conditions is obtained by applying the cloud correction factor to the clear sky emissivity in Equation (9). Inputting the $\varepsilon_{sky}$ and $T_{sky}$ into Equation (7) yields a kinetic canopy temperature that is corrected for the reflected radiation of the background sky. This correction is applied to all the canopy temperature measurements prior to subsequent analyses. The average difference between the IRT measurements before and after emissivity correction over the range of temperatures observed in the field study resulted in an average correction of 1.7 °C.

Equation (8): Sky brightness temperature

(8) $$T_{sky}={\varepsilon_{sky}}^{0.25}*T_{air}$$

Equation (9): Sky emissivity

(9) $$\varepsilon_{sky}=CF+(1-CF)\;*\;\varepsilon_{clear}$$

Equation (10): Cloud correction factor

(10) $$CF=1-\frac{R}{R_{SO}}$$

Equation (11): Clear sky emissivity

(11) $$\varepsilon_{clear}=0.598+0.057*\sqrt{P_{w}}$$

### 2.4. Stem Water Potential

Midday (11:30 to 14:30) stem water potential was measured on six days during the period with the greatest evaporative demand and limited soil moisture (12 August, 24 August, 1 September, 7 September, 9 September, and 14 September 2021). Each day, two stems with mature and fully expanded leaves were collected from nine trees in each of the three irrigation treatments. Prior to sampling, the stems were bagged for at least 20 min in foil-laminated bags (Stem water potential bags, PMS Instrument Co., Albany, OR, USA) to prevent transpiration. Stems were removed from the tree with hand-pruners by cutting the stem below the bagged section, leaving an excised stem protruding from the bag. The bagged, excised stem was then re-cut with a sharp razor blade and, while still in the bag, was placed into a pressure chamber (model 1505D-exp, PMS Instrument Co., Albany, OR, USA). The chamber was pressurized, and water potential (bars of pressure) was recorded when a slight bead of water was visible on the excised stem. The device was calibrated at the beginning of the season by having it serviced at PMS Instruments. We used systematic sampling, collecting data from each treatment group each day but always randomizing the order of the treatment groups from which data were collected and varying the individual trees data were collected from. Data were recorded manually and then entered into an MS Excel spreadsheet. To ensure the accuracy and reliability of the measurements, we checked for leaks in the pressure chamber by using soapy water to look for bubbles in places where there may have been leaks.

### 2.5. Leaf Gas Exchange

Leaf stomatal conductance (gs: H_2_O mol m^−2^ s^−1^) and transpiration (H_2_O mmol m^−2^ s^−1^) measurements were collected with a portable leaf porometer (LI-COR 600, LI-COR Bio. Sciences, Lincoln, NE, USA) on 12 August and 9 September, and both days were sunny and clear. Measurements were collected from three fully expanded, mature leaves that were randomly chosen from each of the nine trees from which stem water potential measurements were collected. Leaves had to be free of any visual defect that could be associated with biotic or abiotic disorders (e.g., yellowing or dark spots). The gas exchange measurements were recorded automatically after clamping the handheld device over each leaf and waiting for the device to stabilize (<30 s). Midday plant measurements captured the maximum stress a tree was experiencing by integrating both above- and belowground environmental factors. The data were stored automatically on the leaf porometer and downloaded at the end of each sampling day onto a computer, where it was stored as an MS Excel file.

### 2.6. Data Processing

Five-minute weather records were averaged over fifteen-minute periods using convolution. Gaps in sensor records that were smaller than 30 min were interpolated linearly. Any periods of time during which there was a known error with the sensor data were removed from the record and substituted with NaN values, and consequently were not included in any aggregations.

A z-score method was employed for outlier detection and removal to prevent the overfitting of the NWSB model and the CWSI to climate anomalies and to enhance the model’s generalizability. The z-score of every data point was calculated for each weather variable. A data point was considered to be an outlier if the z-score had a magnitude greater than 3, based on the empirical rule that 99.7% of data within a normal distribution lie within three standard deviations from the mean [58]. Hence, data points outside this range were statistically significant anomalies and identified as outliers. Days with more than 1 h of consecutive outlier data were not included in the model development. This resulted in two days being removed, June 27 and June 28. These days were part of an unprecedented heat wave across the Willamette Valley, with temperatures greater than 40 °C. Data from this heat wave were excluded from the development of the non-water-stressed baseline and consequently are not included in the CWSI validation results.

### 2.7. NWSB Development

The non-water-stressed baseline (NWSB) was calculated using the midday average canopy temperature data from HZ2 and HZ3 in the well-watered treatment group. The air temperature and RH recorded from the permanent weather station were used to calculate the VPD. The CWSI tends to break down in cloudy weather and during times of leaf wetness [59], because under conditions where the temperature gradient is small, the denominator term of the CWSI tends toward zero. Therefore, days that were cloudy (<300 W/m^2^) or rainy were filtered out for the development of the NWSB model. High wind can also influence canopy resistance and affect CWSI integrity [47,60], so only days with low wind speeds (<1.5 m/s) were used to develop this model. The regression coefficients from this model were used to calculate the aerodynamic and canopy resistance terms using Equations (5) and (6).

### 2.8. CWSI Calculation

The midday climate variables used in CWSI calculation were an average of the data taken at 5 min intervals between 12:00 and 16:00. The midday average values for $\bar{R_{n}}$, $\bar{\rho }$, $\bar{\Delta }$, and $\bar{\gamma }$ for the entire season were 546 Wm^−2^, 1.17 kg m^−3^, 0.216 kPa °C^−1^, and 0.066 kPa °C^−1^, respectively. Combining these parameters and the regression coefficients from the NWSB into Equations (5) and (6) results in aerodynamic and canopy resistance of 52.3 s m^−1^ and 7.5 s m^−1^, respectively. These resistance terms were used to calculate $T_{UL}$ in Equation (4). The average canopy temperature from the well-watered trees (excluding HZ1) was used as the lower-limit temperature ($T_{LL}$). Daily midday averages of T_UL_, T_LL_, T_C_, and T_a_ were then used to calculate the daily CWSI from Equation (1). The calculated CWSI values were validated against actual plant water status measurements, namely, stem water potential (SWP), leaf conductance, and leaf transpiration. The coefficient of determination (R^2^) and the root mean square error (RMSE) were used to assess the accuracy and reliability of the CWSI model.

## 3. Results

### 3.1. Weather Conditions

**Figure 2:**
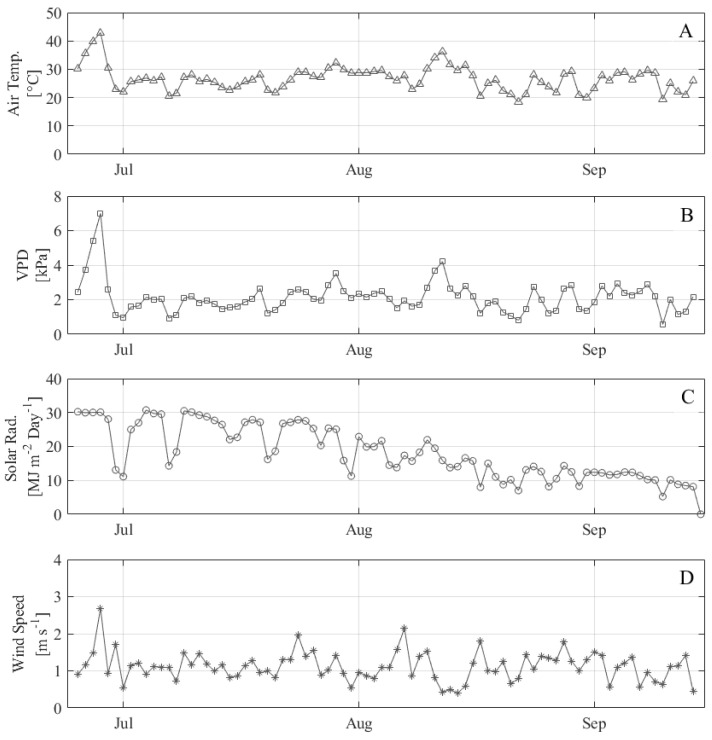
Daily average environmental data at NWREC site from field weather station including (**A**) air temperature, (**B**) vapor pressure deficit, (**C**) incoming solar radiation, and (**D**) wind speed.

The min and max air temperatures over the course of the experiment were 15 °C and 45 °C, respectively (Figure 2). The maximum temperature was observed during an unprecedented heat wave (>40 °C) at the end of June, which resulted in large outliers in the weather data from June 25 to 28. Data from these heat wave days were excluded from the development of the non-water-stressed baseline and consequently are not included in the CWSI validation results.

### 3.2. LOCOS Calibration

**Figure 3:**
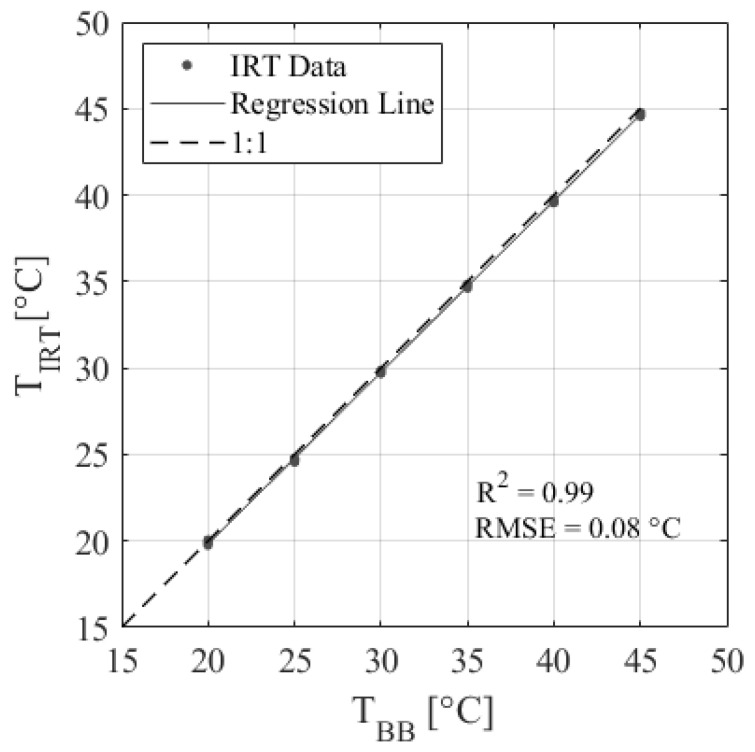
Example of low-cost IRT accuracy showing a linear regression between the IRT object temperature (T_IRT_) and the blackbody calibrator temperature (T_BB_) for IRT 5.

**Figure 4:**
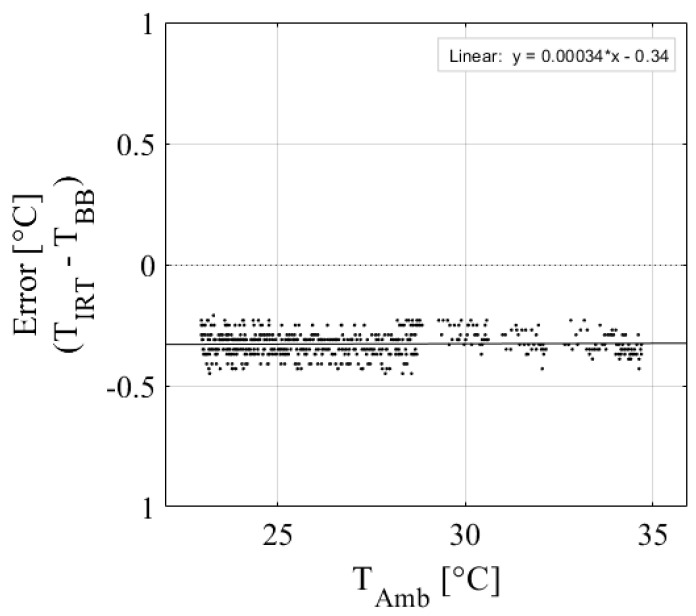
Ambient temperature effect on IRT error for IRT 5, where IRT error is the difference between the blackbody temperature (T_BB_), and the IRT object temperature (T_IRT_).

The low-cost IRT performed well in calibration against a blackbody calibrator. Table 1 shows the calibration results of the nine IRTs, and Figure 3 shows the plotted calibration results of IRT 2. Results show that the IRTs have excellent accuracy, with an average R^2^ = 0.99 and an RMSE = 0.08 °C, which is within the ±0.5 °C accuracy specified by the manufacturer. The regression equations developed are applicable over the range of ambient temperatures observed during calibration (25–50 °C; Table 1). However, IRTs are assumed to respond linearly across all temperatures observed during the experiment (15–45 °C). Figure 4 shows an example of how changes in ambient temperature affect the IRT accuracy for IRT 5. There is a slight but noticeable change in the data distributions between 28 and 35 °C. This is because data from two different calibration days were included to show a wider range of ambient temperatures, so this characteristic is likely due to slight changes in calibration setup. Regardless, the change in IRT error per degree of change in ambient temperature is less than 0.01 °C. Therefore, the effect of ambient temperature on IRT accuracy is assumed to be negligible over the temperature ranges observed in this study.

### 3.3. Crop Water Stress Responses

**Figure 5:**
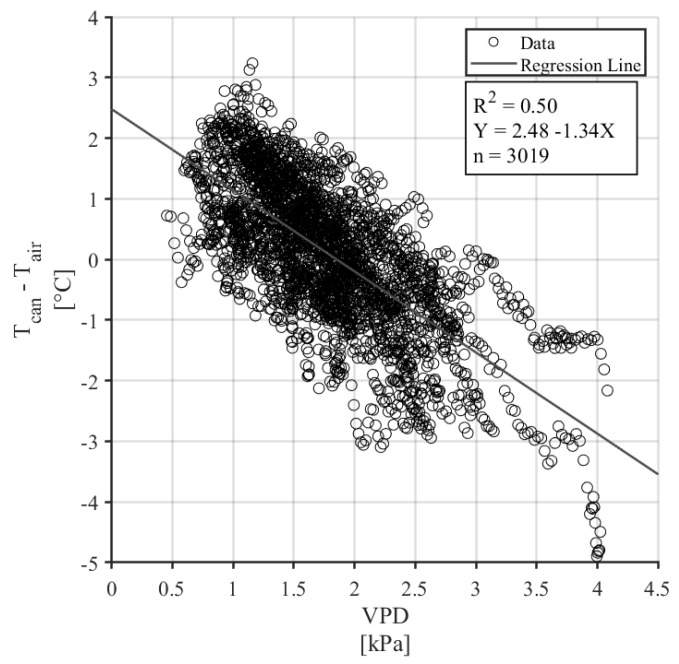
Non-water-stressed baseline with filtered data from well-watered trees (n = 3019).

The T_c_-T_a_ (LTD) of a well-watered canopy was compared to the VPD measured between 12:00 and 16:00, during conditions of full sun, no rain, and low wind speeds (Figure 5). The linear relationship between LTD and VPD was significant with a *p*-value < 0.001 and a coefficient of determination (R^2^) of 0.50. The regression coefficients from this model, 2.48 °C and 1.34 °C kPa^−1^, were used to calculate the aerodynamic and canopy resistance terms using Equations (5) and (6).

**Figure 6:**
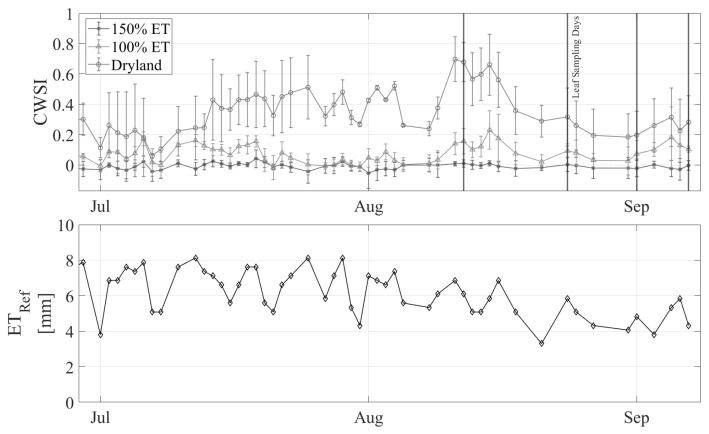
Top—CWSI over the growing season between the three treatments. Bottom—daily reference ET over the season. Vertical lines indicate the days on which SWP samples were taken.

The CWSI was variable throughout the season compared to the daily reference ET (Figure 6). Periods during which the climate variables were outside the bounds set for the NWSB or one of the three LOCOSs for a treatment were not operating are excluded from this plot and the following analyses. The pattern of CWSI values between the three treatments is consistent with what was expected, with the dryland trees consistently experiencing the most stress (largest CWSI), followed by the 100% ET trees, and the 150% ET trees showing the least stress. The maximum CWSI observed in the dryland trees occurred on Aug 11, which was the first day of a three-day heat wave, reaching temperatures greater than 38 °C. The effect of the heat wave was lagged for the 100% ET treatment, with the maximum CWSI occurring three days after the maximum temperature was observed.

**Figure 7:**
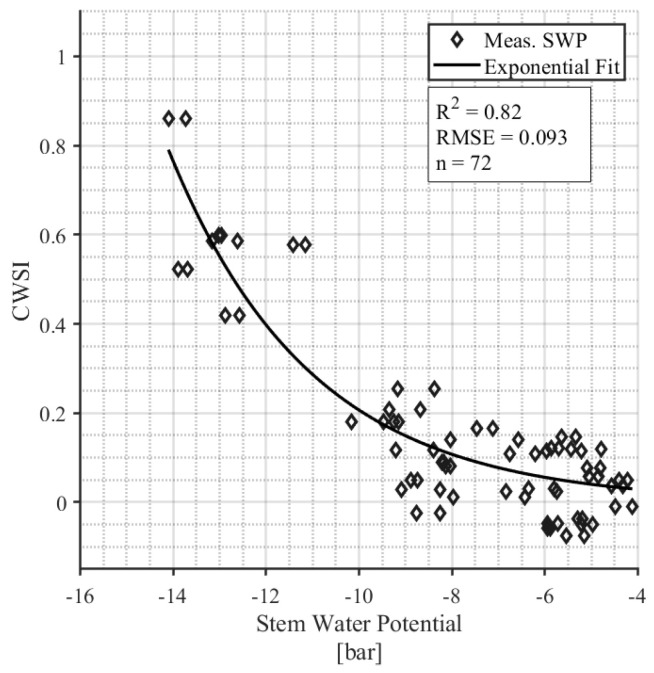
The relationship between CWSI and midday stem water potential measurements.

The daily CWSI was correlated with the stem water potential (SWP) measurements (Figure 7). SWP samples that were taken on days on which the environmental parameters were outside the bounds established for the NWSB were removed from this analysis, resulting in a final sample size of 72 leaves from four survey days (12 August, 24 August, 1 September, and 7 September). The exponential relationship between daily CWSI and SWP is significant (*p* < 0.001) with an R^2^ = 0.83.

**Figure 8:**
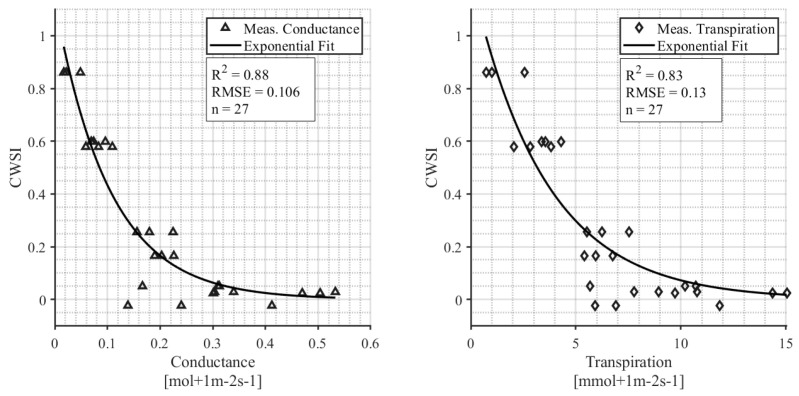
The relationship between CWSI and midday leaf conductance (**left**); and the relationship between CWSI and midday leaf transpiration (**right**).

The relationship between the calculated CWSI and leaf conductance and leaf transpiration is shown in Figure 8. Only measurements from days with weather conditions that were within the bounds specified for the NWSB are included in the analysis, and therefore, the data shown in Figure 8 are from one survey on 12 August 2021. The exponential relationship between the daily CWSI and midday conductance is significant (*p* < 0.001) with an R^2^ = 0.88. The exponential relationship between the daily CWSI and leaf transpiration is significant (*p* = 0.004) with an R^2^ = 0.83.

## 4. Discussion

Our study aimed to investigate the use of low-cost IRT sensors to obtain accurate and repeatable canopy temperature data, facilitating the development of reliable CWSI values for Jefferson hazelnut trees cultivated in Oregon’s Willamette Valley. As a result, our findings yielded two significant themes: first, the validation of low-cost technologies, and second, the establishment of the first-ever CWSI for Jefferson hazelnuts.

### 4.1. Validation of Low-Cost IRT Sensors for Canopy Temperature Monitoring

In the context of the first theme, the validation of the LOCOS, our results indicate that the low-cost IRT sensor demonstrated satisfactory accuracy by effectively detecting differences in tree canopy temperature corresponding to various irrigation treatments (Figure 3, Figure 4 and Figure 5). Calibration further confirmed the sensor’s resilience to changes in ambient temperature within agricultural environments, reinforcing its suitability for agricultural applications. These findings build on previous works that have also demonstrated the potential of the LOCOS in providing irrigation management decisions for high-value horticultural crops [39,61]. In apple trees, another important horticultural crop in the Pacific Northwest, previous research achieved a strong correlation between midday CWSI and stem water potential (R^2^ = 0.91) [17]. Similarly, our study found robust relationships for hazelnuts, with the CWSI correlating with leaf conductance (R^2^ = 0.88) and stem water potential (R^2^ = 0.82). However, the drought tolerance of hazelnuts differs significantly, with stress onset beginning at −10 bar, compared to apple trees’ sensitivity under less severe conditions, highlighting species-specific responses to water deficits.

Incorporating data-driven technologies into agriculture is an effective means of optimizing crop production, particularly in regions that rely on irrigation. By implementing these LOCOSs, farmers can monitor site-specific weather and crop data at a higher spatial resolution to make more informed decisions about crop water use, irrigation timing, and management practices [27]. This is especially important given the increasing frequency of heat waves and droughts associated with climate change [62,63,64]. In arid regions, pomegranates are an important horticultural crop. Previous research on pomegranates demonstrated a strong correlation (R^2^ = 0.976) between a low-cost thermal sensor and a commercial infrared radiometer for the monitoring of canopy temperature in pomegranate trees, validating the sensor’s technical reliability [40]. However, the study focuses solely on comparing sensor outputs without linking temperature changes to physiological indicators of plant water status. In contrast, our work validates low-cost sensors (R^2^ = 0.99; Figure 3) by integrating temperature-based indices like the CWSI with physiological measures, such as leaf conductance and water potential (Figure 7 and Figure 8), to establish meaningful thresholds for plant stress. This approach ensures that temperature readings are directly connected to actionable insights for irrigation management, addressing the critical question of what temperature constitutes “too hot” for effective crop health and production. When assessing new technologies, it is important to consider how experimental designs may impact results, such as the placement of IRTs around the crop canopy.

### 4.2. Challenges in IRT Applications

The optimal orientation of IRT sensors remains a topic of debate, with some researchers advocating for lateral or overhead views, while others support multidirectional placement, such as north and south orientations [40,65,66]. Our IRTs were placed on the north side of the canopy to present a conservative and potentially less noisy measure of crop temperature. Regardless of orientation, precise positioning within the densest area of the canopy is paramount to avoid interference from the horizon, soil, or leafless branches. In addition to sensor performance, challenges persist in accurately recognizing thermal infrared images of plant canopies using traditional image processing technologies. Issues such as strong noise, disturbances, and subtle grayscale differences between the canopy and the background hinder the effectiveness of traditional image segmentation methods, particularly in thermal infrared images. Overcoming these challenges is crucial for advancing the accuracy and efficiency of canopy temperature analysis in agricultural contexts [67]. However, advancements in AI and machine learning, particularly deep learning techniques such as artificial neural networks (ANNs), have shown promise in overcoming these challenges by automatically learning to identify patterns and features in images [14,31]. These techniques have the potential to significantly improve the accuracy and efficiency of image segmentation tasks, including the segmentation of thermal infrared images of plant canopies. AI can aid in traditional thermal imaging challenges associated with IRT placement. The LOCOS may also offer hardware solutions associated with traditional thermal imaging.

The drawbacks of traditional thermal imaging techniques include high camera costs and limited resolution, as well as the significant time and labor required for implementation [25]. Our results add to the growing literature demonstrating that the LOCOS, in this case used with an IRT, can offer a solution to some of the challenges associated with crop stress monitoring at a commercial scale, including low spatial resolution, high cost, and the limited frequency of data collection [68]. By leveraging low-cost technology to increase replication and improve the spatial resolution of ground-based data, researchers and practitioners can develop more robust calibration for drone- and satellite-based crop models. Low-cost modules offer advantages over traditional cameras and UASs, including reduced costs and automatic imaging, but dynamic characterization of the measurement process and a high number of replications are essential for practical applications [40].

### 4.3. Establishing the First CWSI for Jefferson Hazelnuts

Regarding results for the second theme, the establishment of the first-ever CWSI for Jefferson hazelnuts, the regression model correlating canopy temperature difference (Tc-Ta) with vapor pressure deficit (VPD) had an R^2^ of 0.50, indicating that 50% of the variance in Tc-Ta is explained by VPD (Figure 5). While this result is statistically significant (*p* < 0.001), it also highlights that 50% of the variance remains unexplained, likely due to factors such as omitted variables (e.g., microclimatic conditions) and potential noise in the data. This moderate predictive power suggests that using the regression coefficients to calculate aerodynamic and canopy resistance could introduce inaccuracies, particularly under conditions that differ from those in the dataset. Additionally, the context-specific nature of the regression may limit the generalizability of these coefficients, and errors could propagate when these outputs are used in further models. To address these issues, independent data sources are critical for validating the robustness and accuracy of the derived resistance terms.

To evaluate the model further, we incorporated independent measures such as stomatal conductance and stem water potential to test its predictive capability and explore its biological relevance under varying conditions. A strength of the results from our LOCOS is that they were validated against known physiological indicators of water stress including stem water potential, leaf conductance, and leaf transpiration. Stem water potential measurements from pressure chamber instruments are considered to be the gold standard measure of plant water status and are often used as a reference to calibrate models against [13]. The CWSI developed in this study is highly correlated with SWP measurements (R^2^ = 0.84), which indicates that the model can detect real physiological symptoms of water stress in hazelnut trees. Similarly, the CWSI is also well correlated with leaf conductance (R^2^ = 0.74) and leaf transpiration (R^2^ = 0.71), confirming its ability to represent plant hydraulic qualities. These correlations indicate that traditional plant-based irrigation methods can continue to be used by growers in Oregon. The results also clear a path forUAS and remote sensing thermography to be used to incorporate the findings into new models.

We discovered that when SWP is <6 bar, the CWSI is <0.2, and the plants are therefore relatively unstressed (Figure 7). Correspondingly, when the CWSI is <0.2, the leaf conductance rates range from 0.1 to 0.4 mol m^−2^ s^−1^ with a median value of 1.9 mol m^−2^ s^−1^ (Figure 8). Leaf conductance is known to be sensitive to moisture stress in hazelnuts, with other studies reporting that well-watered tree conductance is closer to 0.3–0.4 mol m^−2^ s^−1^ [42]. Our findings support this and suggest that for farmers who use leaf conductance measures, 0.2 mol m^−2^ s^−1^ can be used as an irrigation threshold. One appealing aspect of an IRT compared to handheld leaf conductance measurements is its ability to collect data remotely and continuously. In contrast, handheld leaf conductance measurements are more labor-intensive as they currently rely on manual collection at specific intervals [13]. Continuous measurement throughout the season showed that the un-irrigated “rainfed” hazelnuts had a CWSI > 0.2 from mid-July through the rest of the season (Figure 6). On the other hand, the irrigated hazelnut groups maintained a CWSI < 0.2 for most of the growing season. Our results provide a new benchmark that can be used to compare different production regions and different hazelnut varieties.

### 4.4. Implications of Sensor Technologies and Climate Change for Hazelnut Production

Sensor-based irrigation strategies are critical for hazelnuts because all the major production regions, namely, Turkey, Italy and Spain, and the USA, as well as new regions, namely, South Africa and Australia, have faced historic droughts in recent years. Increasing temperatures worldwide have led to a significant rise in vapor pressure deficit (VPD), a recognized major driver of plant functioning and drought-induced plant mortality [69]. Environmental demand, represented by VPD, interacts with hydraulic traits in regulating plant water use. The observed lag between the dryland trees and the 100% ET trees in response to the August heat wave suggests that the 100% ET trees were able to utilize soil water and plant water storage to delay the effects of heat stress and maintain transpiration longer than the droughted dryland trees, but after prolonged high temperatures and a high VPD, stomata closed, resulting in a noticeable increase in stress. Recent studies have revealed that stomatal conductance in hazelnut trees responds rapidly to induced stress, even if mild, and can be considered an indicator for detecting early signs of stress [42]. Other Mediterranean tree crops have demonstrated a strong relationship between hydraulic traits and the coordination of sap flux density and stomatal conductance in response to water deficit [70]. One study found stand-level canopy conductance (Gsurf) responses to VPD across diverse global sites; however, it did not specify the hazelnut variety [71]. Their findings highlight that hazelnuts maximize their Gsurf at low VPD values (average of 0.57 kPa), suggesting a strategy to avoid leaf dehydration under mild conditions. Our study in Oregon faced consistently higher VPD conditions, with VPD rarely dropping below 1.0 kPa and exceeding 2.0 kPa for much of the growing season (Figure 3). This contrast suggests that hazelnuts may exhibit isohydric tendencies, actively downregulating gas exchange under increasing stress to maintain water potential, but further research is needed to confirm these hydraulic strategies under high VPD conditions.

As climate change leads to more frequent and severe droughts in many regions, hazelnut production could be significantly impacted. Changes in precipitation patterns, increased temperatures, and increased aridity could lead to more water stress in hazelnut trees, especially during critical growth phases [72]. Research has shown that hazelnuts are highly sensitive to water stress, with even slight water stress during sensitive periods significantly reducing vegetative growth, fruit growth patterns, and final yield [43,71,72]. Additionally, a high vapor pressure deficit during dry periods could lead to significant reductions in CO_2_ uptake in hazelnuts, further exacerbating the negative impacts of drought [73]. This inverse relationship is linked to the need to maintain stem water potential above a threshold, which could result in further reductions in CO_2_ uptake and stomatal conductance as soil water in the rooting zone becomes a limiting factor [74]. Due to the limited availability of varied and region-specific data, crop production often relies on generalized theories of plant–environment interactions. For example, the application of a hydraulic framework has proven valuable in providing insights into other agricultural tree crops [75]. Integrating sensors into irrigation decision systems is part of Agriculture 4.0 and requires not only a mastery of technology but also a deep understanding of plant physiology.

## 5. Conclusions

In the era of Agriculture 4.0, LOCOSs demonstrate how the integration of advanced digital technologies holds promise in revolutionizing crop production, particularly in the management of water resources. Our project, focusing on hazelnut production, addressed the pressing need for water resource management in Oregon in one of the hottest years on record. By developing the first CWSI for hazelnuts, which integrated multiple physiological parameters associated with micrometeorology, our study provides an important framework for the understanding of how hazelnut trees respond to weather conditions.

In application with irrigation controllers, the CWSI can optimize water use efficiency, reduce production costs, minimize energy requirements, and enhance nut quality. Sensor-controlled irrigation can be linked with deficit irrigation strategies that have demonstrated a potential for water saving without detriments to hazelnut crop yield [76]. With the diversity of global production regions and new hazelnut varieties, there is great opportunity for more research to be conducted on the CWSI using affordable technologies to move hazelnut production further into the fourth agricultural revolution. The successful validation of low-cost infrared thermometers for Jefferson hazelnut production has led to the development of the first-ever crop water stress index (CWSI) for this cultivar. This provides a novel, data-driven approach to optimizing irrigation in hazelnut orchards, addressing both economic and environmental concerns. By integrating these technologies, growers can effectively manage water resources, enhance crop quality, and potentially mitigate the negative impacts of increasing heat waves and droughts driven by climate change.

## Figures and Tables

**Table 1:** MLX90614 regression coefficients from blackbody calibration, where T__Amb_ Range is the range of ambient temperatures during calibration.

IRT #	Multiplier [°C/°C]	Offset [°C]	RMSE [°C]	R^2^	T_Amb_ Range [°C]
1	1.06	−0.97	0.17	0.99	15.2–30.5
2	1.04	−0.35	0.06	0.99	16.7–27.7
3	1.02	−0.45	0.07	1.00	18.3–34.5
4	0.99	0.52	0.07	0.99	15.3–29.5
5	1.00	0.04	0.08	1.00	16.8–34.7
6	1.00	0.07	0.08	1.00	16.5–25.5
7	1.01	0.06	0.07	1.00	17.5–26.8
8	1.01	−0.15	0.09	1.00	17.4–28.0
9	0.99	0.29	0.07	1.00	19.1–25.7

## Data Availability

Data are contained within the article.
